# Application of Ion Torrent Sequencing to the Assessment of the Effect of Alkali Ballast Water Treatment on Microbial Community Diversity

**DOI:** 10.1371/journal.pone.0107534

**Published:** 2014-09-15

**Authors:** Masanori Fujimoto, Gregory A. Moyerbrailean, Sifat Noman, Jason P. Gizicki, Michal L. Ram, Phyllis A. Green, Jeffrey L. Ram

**Affiliations:** 1 Department of Physiology, School of Medicine, Wayne State University, Detroit, Michigan, United States of America; 2 Center for Molecular Medicine and Genetics, School of Medicine, Wayne State University, Detroit, Michigan, United States of America; 3 Isle Royale National Park, National Park Service, Houghton, Michigan, United States of America; Argonne National Laboratory, United States of America

## Abstract

The impact of NaOH as a ballast water treatment (BWT) on microbial community diversity was assessed using the 16S rRNA gene based Ion Torrent sequencing with its new 400 base chemistry. Ballast water samples from a Great Lakes ship were collected from the intake and discharge of both control and NaOH (pH 12) treated tanks and were analyzed in duplicates. One set of duplicates was treated with the membrane-impermeable DNA cross-linking reagent propidium mono-azide (PMA) prior to PCR amplification to differentiate between live and dead microorganisms. Ion Torrent sequencing generated nearly 580,000 reads for 31 bar-coded samples and revealed alterations of the microbial community structure in ballast water that had been treated with NaOH. Rarefaction analysis of the Ion Torrent sequencing data showed that BWT using NaOH significantly decreased microbial community diversity relative to control discharge (p<0.001). UniFrac distance based principal coordinate analysis (PCoA) plots and UPGMA tree analysis revealed that NaOH-treated ballast water microbial communities differed from both intake communities and control discharge communities. After NaOH treatment, bacteria from the genus *Alishewanella* became dominant in the NaOH-treated samples, accounting for <0.5% of the total reads in intake samples but more than 50% of the reads in the treated discharge samples. The only apparent difference in microbial community structure between PMA-processed and non-PMA samples occurred in intake water samples, which exhibited a significantly higher amount of PMA-sensitive cyanobacteria/chloroplast 16S rRNA than their corresponding non-PMA total DNA samples. The community assembly obtained using Ion Torrent sequencing was comparable to that obtained from a subset of samples that were also subjected to 454 pyrosequencing. This study showed the efficacy of alkali ballast water treatment in reducing ballast water microbial diversity and demonstrated the application of new Ion Torrent sequencing techniques to microbial community studies.

## Introduction

Next generation sequencing techniques have been developed over the last decade such as Roche 454 pyrosequencing [1] and Solexa/Illumina sequencing [2], which enabled us to capture the diversity of microbial communities from various environments [3]–[11]. Amongst next generation sequencing techniques, Roche 454 pyrosequncing has been primarily used for 16S rRNA-based microbial community studies due to its longer average sequence length [12]–[14]. Pipelines such as Ribosomal Database Project (RDP) [15], Mothur [16], and Quantitative Insights Into Microbial Ecology (QIIME) [17] have been developed to analyze pyrosequencing output data. Until recently, the average sequence length obtained with Ion Torrent sequencing was shorter than 250 bp long [14] and was mainly used for clinical studies, such as analyzing mutations in cancer cells [18], cystic fibrosis patients [19], and viral genomes of HIV [20] that did not require longer reads. The few studies that applied the Ion Torrent platform to 16S rRNA gene-based microbial community studies [21]–[23] used 100 or 200 base chemistry and had lower taxonomic resolution than pyrosequencing. However, with the recent release of 400 base chemistry for Ion Torrent sequencing at the beginning of 2013, we were able to design primers for 16S rRNA gene sequencing of up to 410 bp including primer sequences [24], [25], a size comparable to the 454 GS Junior pyrosequencing [25].

In this study, the new Ion Torrent sequencing chemistry was applied to a study of microbial community diversity of ballast water with a focus on the assessment of alkali ballast water treatment efficacy. Ballast water of cargo ships has led to the introduction of many non-native invasive species that have negatively impacted various aquatic ecosystems [26]–[28], as well as the transportation of harmful algae [29], [30] and human pathogens [31], [32]. In order to stop the introduction of non-native species or transportation of harmful bacterial species via ballast water, treatment methods including alkali treatment have been designed to kill the majority of organisms within the ballast tanks before discharge [33].

The objective of this study was to determine the effectiveness of NaOH treatment at pH 12 in reducing microbial diversity and in altering microbial community structure in ballast water using new Ion Torrent sequencing chemistry. To assess the efficacy of the ballast water treatment (BWT), ballast water samples from a Great Lakes ship were collected from the intake and discharge of both control and NaOH (pH 12) treated tanks and were duplicated. In addition, to assess only the viable organisms among the microbial communities in these samples, microbial communities derived from the regular extraction procedure were further compared to duplicate samples that had been treated with propidium mono-azide (PMA). PMA permeates damaged membranes of dead cells and crosslinks the DNA upon exposure to light and makes them not amplifiable during the PCR reaction [34]. Since it was known that only a subset of microorganisms are able to survive in alkaline pH [33], we hypothesized that NaOH would significantly decrease the microbial diversity of the viable microorganisms in the ballast water by eliminating alkali sensitive microbial species.

## Methods

### Ethics Statement

Ballast water samples were collected from the ship *M/V Indiana Harbor* (privately owned by American Steamship Company). We received permission to collect samples from the ship's ballast water from the American Steamship Company. The shipboard sample collection did not involve any endangered or protected species.

### Collection of Ballast Water Samples

Ballast water samples were collected in August 2011 from the ship *M/V Indiana Harbor*, in collaboration with a study by the Great Ships Initiative (GSI). As described in the GSI report [35], the *Indiana Harbor* took on cargo near Duluth, MN and while unloading it in Gary, IN (latitude, longitude  = 41.6147°, −87.3252°, respectively) the ship took on ballast water which was sampled for this study from the intakes to four ballast tanks (2P, 3P, 4P, 5P). Water in two of the ballast tanks (3P and 4P) were treated with NaOH (pH 12) while the ship traveled for 3 days to Superior, WI. The pH of the ballast tanks were adjusted to pH12 by gradually adding 50% (w/v) NaOH into the ballast tanks. The other two tanks (2P and 5P) in the ship served as controls in this study. NaOH-treated tanks were neutralized using an in-tank carbonation system 18 hours prior to the discharge. The ballast water from all four ballast tanks was sampled at the port of Superior, WI (46.7430°, −92.1144°), during the discharge of the neutralized ballast water. A total of 1.0 L of ballast water collected from each tank at each sampling event was kept on ice until initial processing and preservation by methods described below. For the Gary, IN, intake samples initial processing and preservation took place 4 to 8.5 hr after collection from the ship; in Superior, WI, processing and preservation of samples was initiated for all samples within one hr (range 49 to 59 min) of collection.

### Sample processing and DNA extraction

Except as noted in summary Table 1, four aliquots of 100 mL each were dispensed from each 1 L sample and processed with either PMA (two aliquots) or non-PMA methods (two aliquots). The 100 mL aliquot of non-PMA group was filtered using a 0.22 µm syringe filter (Thermo Nalgene cat. no. 190–2520). For PMA assay, the 100 mL aliquot of the PMA group was filtered with 0.22 µm filter membrane, and 0.8 mL of 100 µM PMA was applied to the filter associated microbes. The filter associated microbes were incubated in the dark for 15 minutes to allow PMA to bind to the DNA of dead or injured cells. Then, the microbes on the filter were exposed to an intense white LED light (Husky 180 LED Work light, Home Depot cat. no. 955–998) for 7 minutes to allow PMA to cross-link the DNA [34]. The filter associated microbes from both PMA and non-PMA groups were backwashed off the filter and preserved with 1 mL of DNAzol Direct (Molecular Research Center, cat. no. DN 131). The DNAzol Direct sample solutions were transported to Wayne State University and stored at −80°C. To purify the genomic DNA, 230 µL of DNAzol Direct sample solutions were purified using Qiagen spin column purification kits (Qiagen, cat. no. 69506), according to the manufacturer's protocol.

**Table 1:** Experimental design of alkali ballast water treatment.

Ballast Treatment	Ballast Tank ID	Intake		Discharge	
		Non-PMA	PMA	Non-PMA	PMA
Control	2P	35N, 40N	25P, 30P	53N, 57N	45P, 49P
	5P	36N, 41N	26P, 31P	54N, 58N	46P, 50P
NaOH	3P	37N, —42N*	27P, 32P	55N, 59N	47P, 51P
	4P	38N, 43N	28P, 33P	56N, 60N	48P, 52P

Sample labels are shown for each 100 mL aliquot of ballast water samples collected at intake and discharge from both the control and NaOH treated ballast tanks. Duplicate aliquots from each ballast water sample were processed with and without propidium mono-azide (labeled ##P and ##N, respectively).

*Sample 42N was spilled at the initial processing site and therefore not available for Ion Torrent sequencing.

### Ion Torrent Sequencing

The library for Ion Torrent sequencing was prepared by amplifying a 410 bp segment of the V4-V5 region of 16S rRNA gene [36], using U515F (5′-GTGCCAGCMGCCGCGGTAA-3′) [10], [37] and U926R (5′-CCGTCAATTCMTTTRAGT-3′) [8], [38] primers. The V4 and V5 regions have great sequence variability and are sensitive enough to identify diverse groups of bacterial taxa [10], [39]. This primer set is also predicted to amplify a wide range of microbial taxa by blast of public sequence databases. The primer set was also confirmed experimentally using DNA extracts of pure culture on *E. coli*, *Vibrio cholera* and intestinal enterococci, named in various proposed and adopted ballast water regulations [40], [41]. The forward primer was linked with the Ion adapter “A” sequence (5′-CCATCTCATCCCTGCGTGTCTCCGACTCAG-3′) and Ion-Xpress barcode sequences were inserted between the adapter “A” and the U515F primer. The reverse primer was linked to Ion adapter “p1” sequence (5′-CCTCTCTATGGGCAGTCGGTGAT-3′).

PCR amplification was performed in 50 µL reactions, using 2 U of high fidelity DNA polymerase AccuPrime Taq HiFi (Invitrogen, Grand Island, NY), 5 µL of supplied 10X buffer II, 1.0 µL of 10 µM primers, and 3 µL of template DNA. The PCR protocol was denaturation at 95°C for 5 min followed by 32 cycles of denaturation at 95°C for 30 s, annealing at 55°C for 30 s, and extension at 72°C for 2 min. The extension of 2 min was employed to minimize the formation of short fragments and chimeras [38], [42]. Thirty-two cycles was used so that the PCR amplification was stopped at the pre-saturation point (the saturation point was pre-assessed by running real time PCR with SYBR green under the same PCR condition).

After purifying PCR amplicons using Agencourt AMPure XP Beads with 1.5X concentration (Beckman Coulter, Inc., Brea, CA), the concentrations of the purified PCR products were measured using PicoGreen (Life Technology, NY), and a library mix was prepared by adding an equal mass of DNA from each barcoded sample. The amplicon size and concentration of the library mix was confirmed using TapeStation 2200 (Agilent Technologies, Inc) with high sensitivity D1K screen tape and reagents. The library mix was diluted with ultra-pure water to 26 pM. The template preparation for the PGM sequencer was prepared from 25 µL of the 26 pM solution using a One Touch 2 (OT2) system (Life Technology, Inc) according to the manufacturer's protocol. Each PCR amplicon was hybridized on an ion sphere particle (ISP) and amplified clonally in the OT2. The clonally amplified ISPs were re-suspended and enriched manually by selecting for ISPs with sufficient amplification using Dynabeads (Life Technology, NY). Three µL of the enriched ISPs were mixed with 3 µL of PGM primers and annealed in a thermocycler with 95°C for 2 min followed by 37°C for 2 min. One µL of DNA polymerase was added to the 6 µL template ISPs-primer mix, and a total of the 7 µL mix was pipetted and added into an Ion 314 V2 chip. The mix was equally distributed across the chip by pipetting up and down, so that in principle each well in the 314 chip was filled with only a single ISP. The ISP-loaded 314 chip was mounted in the Ion PGM sequencer and DNA on the ISPs was sequenced with 800 flows after pH of the PGM system was adjusted. Two PGM sequencing runs were performed: the first run with 16 samples plus one positive control (*Vibrio cholera* JW612) and the second run with 15 samples plus two positive controls (*V. cholera* JW612 and *Enterococcus* sp.). The combined data from the two PGM runs were analyzed.

### Ion Torrent data analysis

Ion PGM output were generated with binary alignment map format (bam) and converted to standard flowgram format (sff) using the “bam2sff” function. The resultant sff file was converted to fasta (sequence), flow (flowgram), and qual (quality score) files using Mothur version 1.30.00 (released April 2013). The fasta and qual files were processed using Ribosomal Database Project (RDP) pipeline version 10 [43] to sort the sequence data by barcodes, to trim barcode and forward primer sequences, and to filter out low quality sequences, retaining only sequences with quality scores above 20 (probability threshold of 0.01) and read lengths of >250 bp. The RDP processed (filtered/trimmed) fasta files were concatenated into a single fasta file and each sequence was transformed into valid QIIME format. Chimeric sequences were identified using the Uchime reference algorithm [44] and removed from the subsequent analysis (0.8% sequences were removed in this process). Alpha and beta diversity analyses of the ballast water samples were performed using a QIIME pipeline [17]. The QIIME formatted sequences were clustered at 97% similarity cut off, and the taxonomy of each cluster was assigned using RDP classifier at a bootstrap threshold of 70%. Rarefaction curves of the OTUs with 97% similarity cutoff were generated using the alpha rarefaction.py command in QIIME, and the effect of NaOH treatment on the microbial community diversity (number of OTUs) was assessed using a general linear model after rarefying all samples at 11,000 reads [45]. Other diversity indices including Chao1 and Shannon were also calculated using the same command. The general linear model was performed using “lm” function in R version 3.0.0 [46]. The treatment effect was also assessed using multiple comparison tests within ANOVA. P-values were controlled for multiple comparisons for *posteriori* hypotheses with Tukey's honest significant difference (HSD). The ANOVA and multiple comparisons with Tukey's HSD were performed using the “multicomp” package in R version 3.0.0 [46]. To assess the beta diversity between different treatment groups of ballast water samples, weighted UniFrac distances [47] between samples were calculated after rarefying all samples at 10,000 reads. The effect of alkali water treatment on ballast water microbial communities was depicted using UniFrac distance based principal coordinates analysis (PCoA) plots [48] and UPGMA tree [49]. To assess the statistical significances in microbial community dissimilarity between particular treatment groups, the Unifrac distance matrix generated in QIIME was transferred into R and ANOSIM was performed in R with the vegan package. Correlation between microbial community samples were also examined by Pearson's correlation in R using BIOM matrix data (with 97% similarity cutoff) generated in QIIME after rarefying at 10,000 reads. Ion Torrent sequence data obtained from this project is available at the NIH Sequence Read Archive (SRA) under the project accession number SRP042367.

### Genus *Alishewanella* phylogenetic tree

A genus-level phylogenetic tree for *Alishewanella* reads was constructed in MEGA version 5.2 [50]. 16S rRNA gene reference sequences of all 6 known *Alishewanella* species plus some of *Alishewanella* sp. identified at the genus level were downloaded from the NCBI database and aligned with 102 randomly selected Ion Torrent reads from sample 47P (one of the NaOH treated discharge samples). The phylogenetic tree was constructed using the Neighbor-Joining algorithm [51] with the Maximum Composite Likelihood method [52]. A total of 357 positions (alter eliminating gaps with complete deletion) were used to calculate the distances for the Neighbor-Joining tree. At each node, bootstrap values with 100 replications were placed based on the frequency that the sequences appeared in the same cluster.

### Pyrosequencing and Data Analysis

The Ion Torrent sequence data were compared to pyrosequencing data obtained in a pilot study on a subset of the samples (controls 26P,31P, 46P, 50P; and NaOH treatment 28P, 33P, 48P, and 52P samples) chosen from Table 1. The selected samples were PCR-amplified with barcoded universal primers targeting the V1-V2 region of the 16S rRNA gene using Adapter/27F (5′ - GCCTTGCCAGCCCGCTCAGT CAGAGTTTGATCCTGGCTCAG - 3′) and Adapter/*Barcode*/338R (5′ -GCCTCCCTCGCGCCATCAG *NNNNNNNN* CATGCTGCCTCCCGTAGGAGT- 3′) [53] primers. The PCR products were gel purified using a Qiagen gel purification kit (Qiagen, cat. No. 28704), concentrations of the PCR products were measured using NanoQuant (Thermo Fisher Scientific Inc, NH), and then pooled in approximately equimolar amounts. The pooled sample was sequenced by pyrosequencing on a 454 Life Science Genome Sequencer FLX (Roche) at the Environmental Genomics Core at the University of South Carolina, Columbia. Resultant sequence data in standard flowgram format (sff) format were processed using RDP pipeline version 10 [15] to sort the sequence data by barcodes, to trim barcode and forward primer sequences, and to filter out low quality sequences with minimum quality score of 20 (probability threshold of 0.01). The minimum read length was set at 150 bp. The resultant pyrosequence data were compared to those obtained using Ion Torrent sequencing by employing the RDP multiclassifier with the bootstrap threshold set at 60% [54]. Bray-Curtis dissimilarity based PCoA plot was generated using the output of the RDP multiclassifier. Bray-Curtis dissimilarity matrix was generated with the “vegan” package in R using the genus level OTUs matrix that had a relative abundance of 0.5% or more (to remove the effect of singletons and doubletons). PCoA plots were generated in R using the “labdsv” package. The correlation in microbial communities between those derived from Ion Torrent and Pyrosequensing were assessed by applying Pearson correlation analysis to the output of the RDP multiclassifier in R. Pyrosequencing data obtained from this project is available at the NIH Sequence Read Archive (SRA) under the project accession number SRP042367.

## Results

After filtering/trimming Ion Torrent output data, a total of 580,605 quality reads were obtained, with an average number of reads equal to 18729±4610 reads and an average sequence length of 380±3 bases per sample, as summarized in Table S1. Purified DNA from a pure culture of *Vibrio cholera* JW612, used as a control for each Ion Torrent run, generated 16270 *Vibrio* sequences out of 16280 reads (99.94%) in the first run and 28031 out of 28082 reads (99.82%) in the second run.

**Figure 1:**
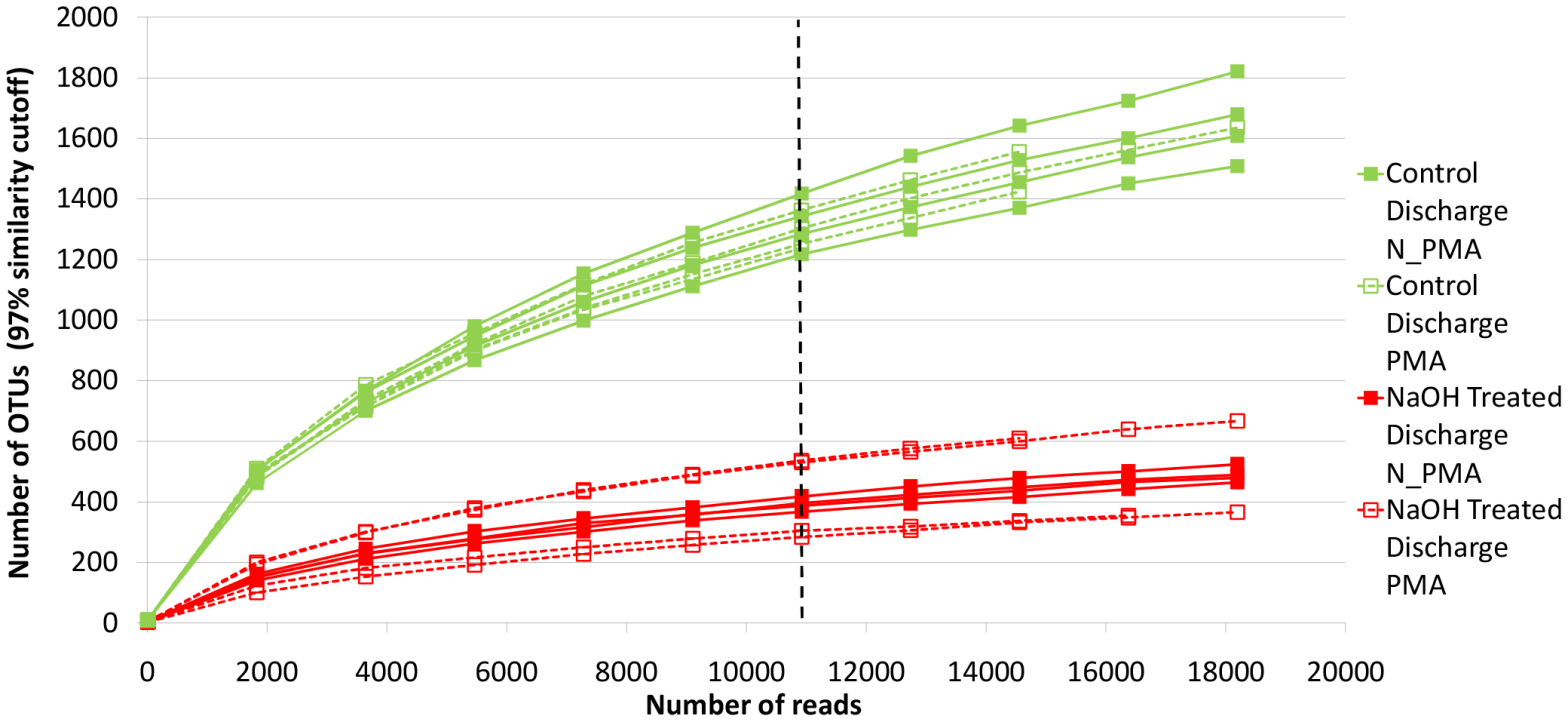
The rarefaction curve of Ion Torrent sequence data. OTUs were defined at 97% similarity cutoff. The figure depicts the comparison between control discharge and NaOH treated discharge samples. The breaking line was placed at around 11,000 (10,918) reads and OTUs were compared across samples when samples were rarefied at 11,000 (10,918) reads.

Rarefaction curves of samples shown in Figure 1 and Figure S1 indicate that none of the samples reached saturation, but it appears that the NaOH-treated discharge samples are closest to being saturated. The number of OTUs defined at 97% similarity cutoff were rarefied at around 11,000 reads per sample and were compared across samples (Figure 1 and Figure S1). NaOH treated discharge samples had an average 403 OTUs, which was significantly lower than the number of OTUs in NaOH intake samples (average OTUs = 907, t_27_ = 7.102, p<0.001) and control discharge samples (average OTUs = 1302, t_27_ = 13.11, p<0.001). The number of OTUs of control discharge increased slightly compared to intake water to control tanks (average OTUs = 1101, t_27_ = 2.933, p = 0.0067). The statistical significance of the above test held after accounting for multiple comparisons using Tukey's HSD (p = 0.032). Similar results were observed for other diversity indices as well (Table 2). For more detailed information, a summary of diversity measurements for each sample is available in Table S2.

**Table 2:** Summary of averaged diversity measurements for each treatment group rarefied at 10,918 reads.

	Number of OTUs	Chao1	Shannon
Control Intake	1101	1764	7.226
	(199)	(354)	(0.300)
NaOH Intake	907	1457	6.761
	(152)	(265)	(0.292)
Control Discharge	1302	2101	7.873
	(69)	(158)	(0.132)
NaOH Discharge	403	670	2.971
	(92)	(159)	(0.813)
NaOH Discharge vs. Control Discharge	p<0.001	p<0.001	p<0.001
NaOH Intake vs. NaOH Discharge	p<0.001	p<0.001	p<0.001
Control Intake vs. Control Discharge	p = 0.007	p = 0.011	p = 0.010
Control Intake vs. Control Discharge (HSD)	p = 0.032	p = 0.051	p = 0.045

The numbers inside the parentheses represent the standard deviation. The bottom half shows results from a statistical test comparing treatment groups of interest for each diversity index.

**Figure 2:**
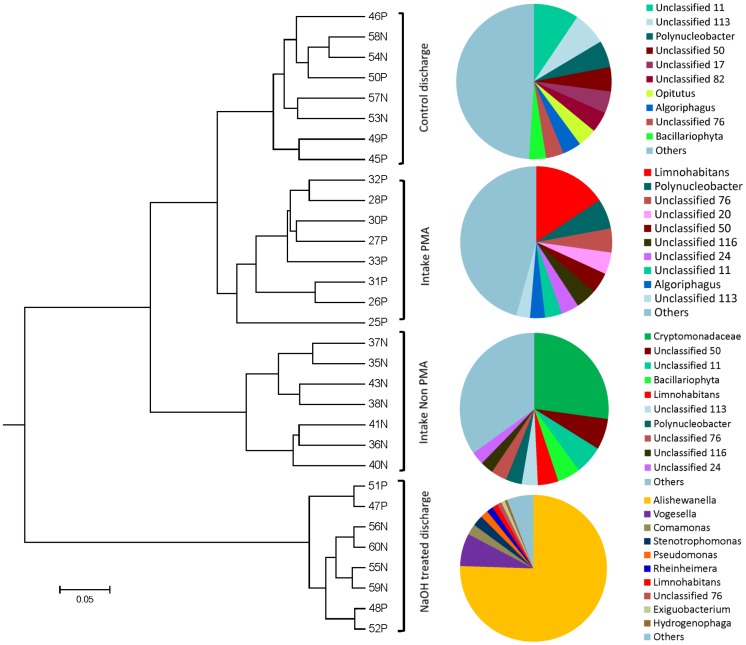
A UPGMA tree constructed using weighted UniFrac distance matrix among the ballast water samples. “P” and “N” in sample ID denote for PMA processed and Non-PMA processed, respectively. The Pie charts depict microbial community structure at genus level for each group. The top ten most abundant genera were shown and others were lumped in others.

16S rRNA gene amplicon sequence data analysis revealed that microbial community structure characterized at the genus level was altered by NaOH treatment (Figure 2 and Figure S2). Microbial communities of PMA-processed intake samples were similar to one another, with *Limnohabitans*, a genus of beta-proteobacteria, being particularly prominent averaging 15% of the relative abundance across PMA processed intake samples. Intake samples processed without PMA were similarly consistent across all intake samples, with *Limnohabitans* clearly present and various Cryptomonodaceae being more prominent than in the PMA-processed samples. In contrast, *Alishewanella*, a genus of gamma-proteobacteria, accounted for over 50% of the community in NaOH-treated discharge samples. A neighbor-joining tree of *Alishewanella* reads revealed that they were not homogeneous, but had variability in sequences which encompass 3 previously known *Alshewanella* species (Figure S3). The relative abundance of other gamma-proteobacteria genera such as *Rheinheimera* and *Pseudomonas* also increased as well as genera of the phylum Firmicutes such as *Bacillus* and *Exiguobacterium*. Bacteria from the genera *Escherichia*, *Enterococcus*, and *Vibrio* were not detected above noise level (i.e. singleton or doubleton) in either intake or discharge samples in this study. Weighted UniFrac distance based principal coordinate analysis (PCoA) (Figure S4) and UPGMA tree (Figure 2) revealed that the microbial communities treated with NaOH were different from those of intake samples and control discharge samples, and the differences were statistically significant when assessed using Unifrac distance based ANOSIM (p = 0.001 and p = 0.001 for NaOH discharge vs. all intake samples, and NaOH vs. Control discharge, respectively). Correlation analysis also revealed that microbial communities of NaOH treated discharge samples did not correlate with those of other groups (Table S3). A small difference in microbial community structure between intake and discharge of control tanks was present; however, this difference was smaller than that between intake and discharge of NaOH treatment tanks (Unifrac distance in Figure 2).

The viable portion of microbial community was assessed by processing samples with PMA. Intake community structures differed between PMA-processed (i.e. live) and non-PMA processed (total DNA) duplicates as described above (Figure 2 and Figure S2). This difference between PMA and non-PMA processed intake samples was also detected in the PCoA plots (Figure S4) and the UPGMA tree (Figure 2), and the ANOSIM showed that the difference was significant (p = 0.001). In contrast, discharge samples exhibited no significant difference in the microbial community structure between PMA and non-PMA processed samples (p>0.10 for both control and NaOH discharge, based on ANOSIM).

**Figure 3:**
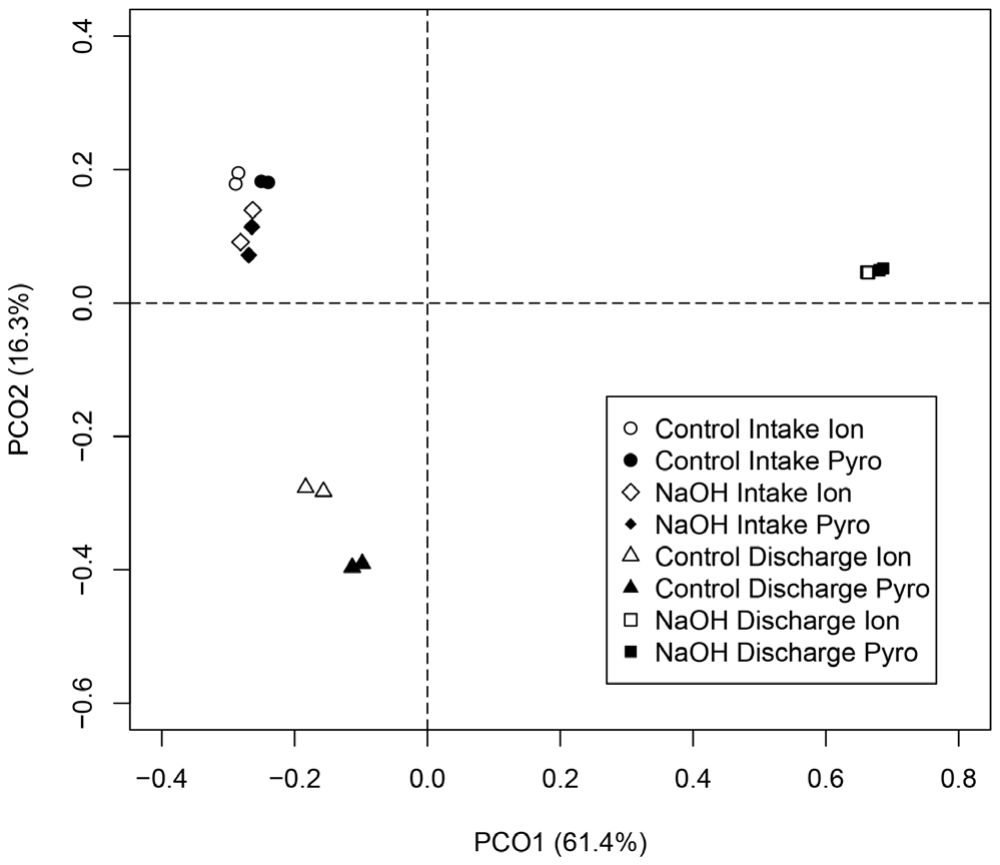
PCoA plot depicting a comparison of microbial communities derived from Ion Torrent and pyrosequencing. RDP multiclassifier with 60% threshold was used to identify microbial taxa in each sample at genus level. PCoA plot was generated using Bray-Curtis dissimilarity matrix. Taxa that had the relative abundance of 0.5% or greater at least one of the samples were used to generate the matrix. The values in the parentheses indicate the percentage of eigenvalues for each axis.

The microbial community structures obtained using Ion Torrent sequencing were compared to those obtained in a pilot study using pyrosequencing on a subset of the samples (Figure 3 and Figure S5). Substantial correlation in microbial community structure occurred between Ion Torrent sequence data and pyrosequence data when compared at the genus level using Pearson correlation analysis (Table S4).

## Discussion

This study demonstrated the application of new Ion Torrent sequencing chemistry to the assessment of microbial community compositions and diversity. Ion Torrent sequencing generated a comparable number of sequence reads and displayed similar microbial community structure to that obtained in a pyrosequencing pilot study. Ion Torrent sequencing also proved to be accurate based on sequences generated in controls. Although the primer set used in the Ion Torrent analysis is targeted at a different region of 16S rRNA genes than the primers used for pyrosequencing, it nevertheless detected a similarly wide range and similar community assembly of microbial taxa. Many of the genera identified in these samples were the same with both methods, but at much lower cost and faster speed with the Ion Torrent.

Alkali ballast water treatment reduced microbial community diversity as shown with OTU richness and other diversity indices and altered microbial community assembly as hypothesized. The most notable change was the increase in the relative abundance of gamma-proteobacteria genera, including *Alishewanella*, *Rheinheimera*, and *Pseudomonas*, and the decrease in beta-proteobacteria including the genus *Limnohabitans* after the alkali treatment. *Alishewanella* strains were isolated from various environments including an Alkali pond in Nebraska (*Alishewanella* sp. SG13. NCBI#:HQ413096), a human fetus [55], tidal sediments [56], fermented seafood [57], landfills [58], textile dye contaminated soils [59], and industrial effluents [60]. According to a previous study by Kim et al. 2010 [58], *Alishewanella agri* BL06 can grow in the pH range between 5.5 and 12.0. Previous studies also found that genus *Alishewanella* is closely related to genera *Rheinheimera* and *Alkalimonas* [57], [61], the latter of which is known to prefer alkali conditions for the optimum growth [61]. The fact that *Alishewanella* spp. accounted for over 50% of relative abundance in ballast water microbial community after NaOH treatment suggests that some strains of *Alishewanella* are especially resistant to alkali stresses.

A previous study that tested alkali resistance among microbial species reported that 11 species of gamma-proteobacteria that they tested were all sensitive to alkali treatment [33]. However, the present study detected a significant increase in the relative abundance of gamma-proteobacteria genera of which the most prominent example was the genus *Alishewanella*. This different outcome could be due to the different experimental setting between the studies. While the previous study was performed using cultures grown in broth (thus, planktonic phase), we tested the efficacy of the alkali water treatment using real ballast water tanks where biofilm could be formed on the surfaces of the tanks [62]. Some gamma proteobacteria strains including *Alishewanella* sp. [63] and *Pseudomonas* sp. [64] are known to form biofilm. Possibly, these gamma-proteobacteria strains survived alkali treatment in the ballast tanks by forming biofilm, which is known to confer resistance to environmental stresses [65]. This suggests that studies on microbial alkali resistance with planktonic phase in laboratory setting may not apply to the real ballast water tank scenario.

Starliper et al. [33] also reported that Gram positive microorganisms, such as those belonging to the phylum Firmicutes, were more resistant to alkali stresses than other phyla they tested. The present study found a similar trend in that the relative abundance of *Bacillus* spp. increased after NaOH treatment. Although this increase in a Firmicutes genus with alkali treatment was detected, these organisms were a small fraction of the total community, accounting for less than 1% of the relative abundance.

Microbial diversity slightly increased from control intake to control discharge. These additional taxa could be derived either from sediments of the ship tank [66] or surface of the tank [62]. Since additional taxa were added during the voyage, the efficacy of the NaOH ballast water treatment should be assessed by comparing control discharge and NaOH discharge rather than NaOH intake and NaOH discharge. Despite the increase in diversity over time in the control tanks, microbial diversity nevertheless significantly decreased when the tanks were treated with NaOH.

The PMA vs. non-PMA comparisons showed that cyanobacteria/chloroplast16S rRNA gene sequences were present in intake samples, but were mostly present in PMA-permeable dead or dying cells. This suggests that the Gary, IN harbor may have had a recent algal bloom in August 2011, although no independent report has been made regarding a bloom around that time. In contrast to this apparent live-dead difference in community in environmental samples, after 3 days treatment of water in ballast tanks, no community assembly differences were present between PMA and non-PMA samples. This was possibly because the genomic materials of dead organisms degraded by the time the ballast water samples were collected.

The new Ion Torrent sequencing chemistry generated data comparable to pyrosequencing. Community structures derived from these two different sequencing techniques were congruent. Both methods have a higher likelihood of errors when homopolymer nucleotides are present since both techniques employ one nucleotide at a time without termination and are thus sensitive to insertion and deletion (in/del) errors [25], while Illumina sequencing is less affected by homopolymer issue [25]. The error rate of Ion Torrent sequencing with the new 400 base chemistry was found to be comparable to that of pyrosequencing [25].

Although our study suggests that some genera of gamma proteobacteria survived through alkali treatment, only two ballast tanks were treated in this study, which is an insufficient sample size for generalization. NaOH treated ballast tanks from other ships need to be studied before the result of this study can be generalized.

The sodium hydroxide treatment adjusted at pH 12 managed to significantly reduce the microbial species diversity of the ballast water. However, it did not manage to completely eliminate all viable microorganisms. Concerns remain with regard to the strains resistant to the alkali treatment. In our study, we focused on the effect of alkali treatment on taxa diversity; however, it is desirable to not only decrease the number of taxa but also to reduce the viable microbial quantity to minimize the impact of biota transportation via ballast water. The Great Ships Initiative (GSI) measured the microbial quantity using heterotrophic most probable number (MPN), and found that the microbial quantity of NaOH treated discharge was two orders of magnitude greater than that of the control discharge [35]. GSI concluded that this was probably due to either the sloughing off of biofilm formed on the inside of ballast tanks or sediments, and/or re-growth of bacteria that were resistant to the alkali treatment after the neutralization. The protective mechanisms of surviving bacteria in the treated tanks (i.e. such as biofilm formation) need to be analyzed and the timing of the neutralization before discharge needs to be evaluated in order design a more effective treatment. A multi stage treatment combining other chemical and ultraviolet light might be more effective at removing organisms in the ballast tanks. Alternatively, the ballast water treatment could target more specific groups of microbes that are known to be harmful and/or pathogenic.

## Supporting Information

Figure S1 **The rarefaction curve of Ion Torrent sequence data.** OTUs were defined at 97% similarity cutoff. Panel A) displays the comparison between NaOH intake and NaOH treated discharge samples, and panel B) shows the comparison between control intake and control discharge samples. The breaking line was placed at around 11,000 (10,918) reads and OTUs were compared across samples when samples were rarefied at 11,000 (10,918) reads.
(TIF)

Figure S2 **Microbial community assembly of ballast water samples determined at genus level using Ion Torrent.** Taxa that have the relative abundance of 2.5% or greater in at least one of the samples were shown in this figure. Some dominant genera were annotated on the figure. “P” and “N” in sample ID denote for PMA processed and Non-PMA processed, respectively. Genus *Bacillus* was included although its relative abundance did not exceed 2.5% in any samples.
(TIF)

Figure S3 **A genus level phylogenetic tree of** ***Alishewanella*** **reads.** The tree was constructed using Neighbor-Joining algorithm. *Rheinheimer*a and *Alkalimonas* were used as out-group. 102 *Alishewanella* reads were randomly selected from sample 47P and aligned with *Alishewanella* reference sequences obtained from NCBI. The number in the parentheses represents the number of 47P *Alishewanella* reads in the respective clade.
(TIF)

Figure S4 **Principal coordinate analysis (PCoA) plots of the ballast water samples using weighted UniFrac distance.**
(TIF)

Figure S5 **Comparison of microbial community assembly derived from Ion Torrent and pyrosequencing.** RDP multiclassifier with 60% threshold was used to identify microbial taxa in each sample at genus level. Taxa that had the relative abundance of 1.5% or greater at least one of the samples were included in this figure.
(TIF)

Table S1 The number of reads and the average size obtained using Ion Torrent sequencing with the 400 chemistry for the 31 ballast water samples.
(PDF)

Table S2 Summary of diversity measurements for each sample rarefied at 10918 reads.
(PDF)

Table S3 Pearson correlation analysis for Biom matrix data.
(PDF)

Table S4 Pearson correlation analysis for the RDP multiclassifier output.
(PDF)
