# Supplementary figures and images for: Application of Ion Torrent Sequencing to the Assessment of the Effect of Alkali Ballast Water Treatment on Microbial Community Diversity
Source: PLoS One. 2014 Sep 15;9(9):e107534. doi: 10.1371/journal.pone.0107534 (PMC4164647; doi:10.1371/journal.pone.0107534)

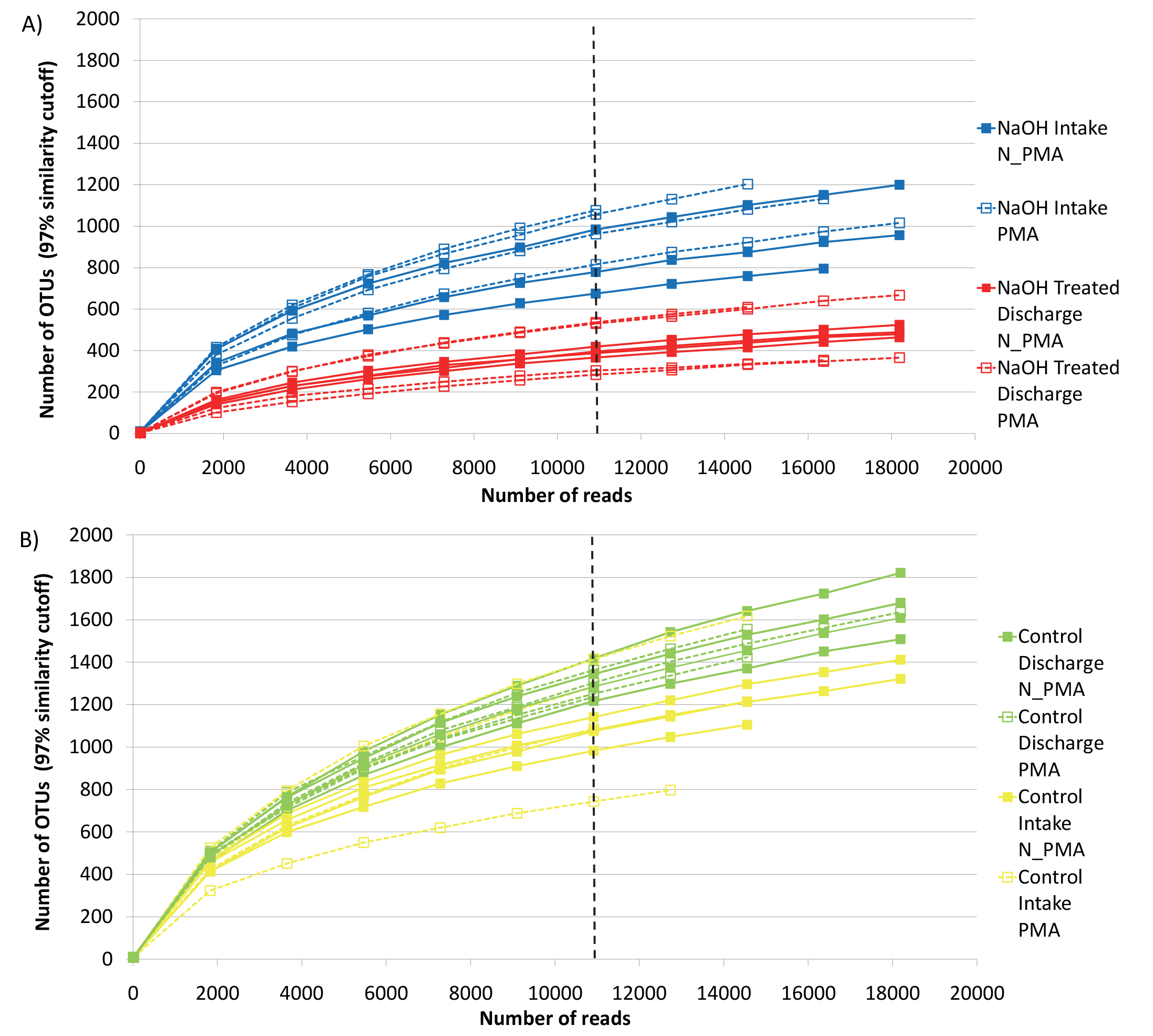

Supplement: Figure S1 — The rarefaction curve of Ion Torrent sequence data. OTUs were defined at 97% similarity cutoff. Panel A) displays the comparison between NaOH intake and NaOH treated discharge samples, and panel B) shows the comparison between control intake and control discharge samples. The breaking line was placed at around 11,000 (10,918) reads and OTUs were compared across samples when samples were rarefied at 11,000 (10,918) reads. (TIF) [file pone.0107534.s001.tif]

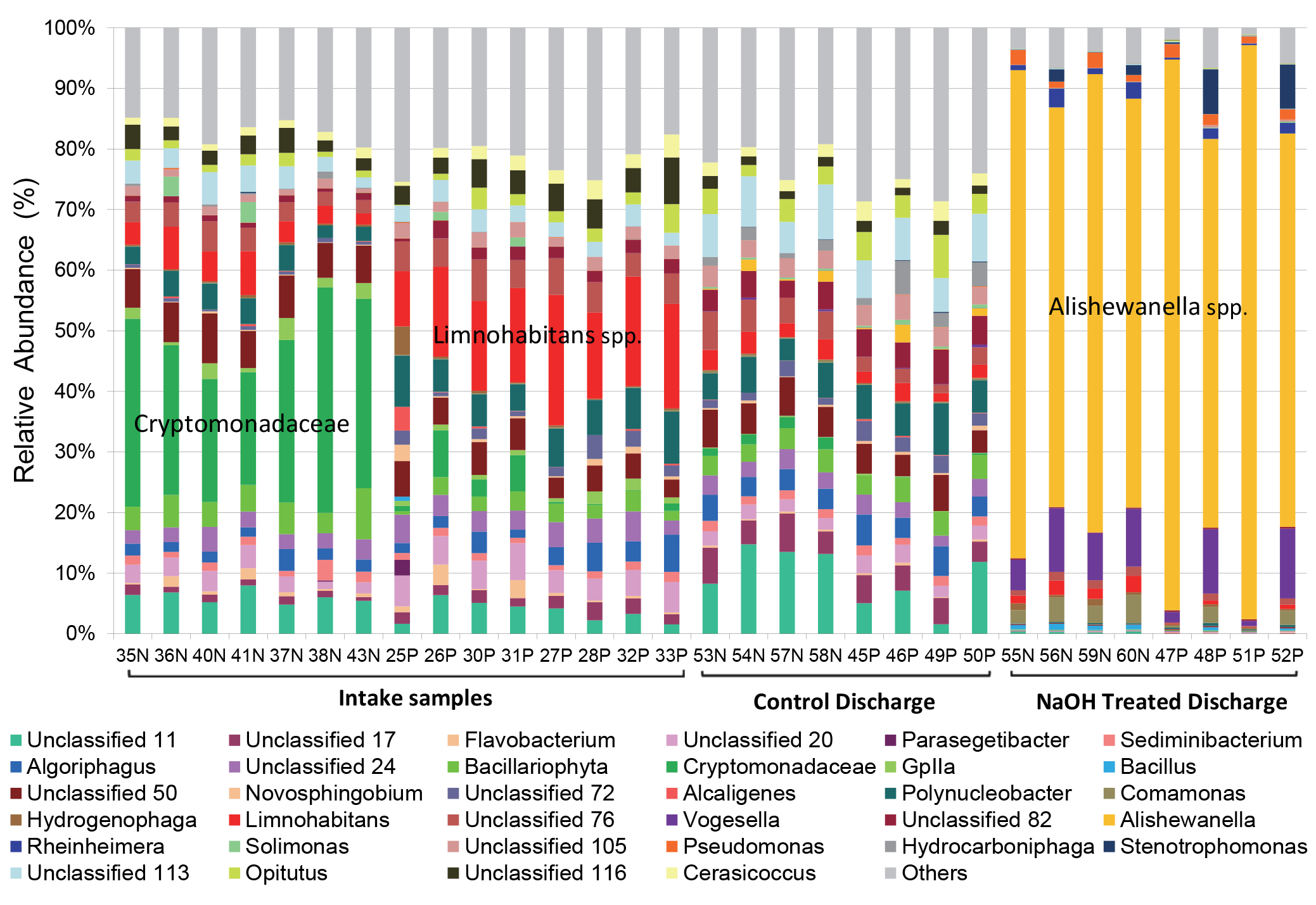

Supplement: Figure S2 — Microbial community assembly of ballast water samples determined at genus level using Ion Torrent. Taxa that have the relative abundance of 2.5% or greater in at least one of the samples were shown in this figure. Some dominant genera were annotated on the figure. “P” and “N” in sample ID denote for PMA processed and Non-PMA processed, respectively. Genus Bacillus was included although its relative abundance did not exceed 2.5% in any samples. (TIF) [file pone.0107534.s002.tif]

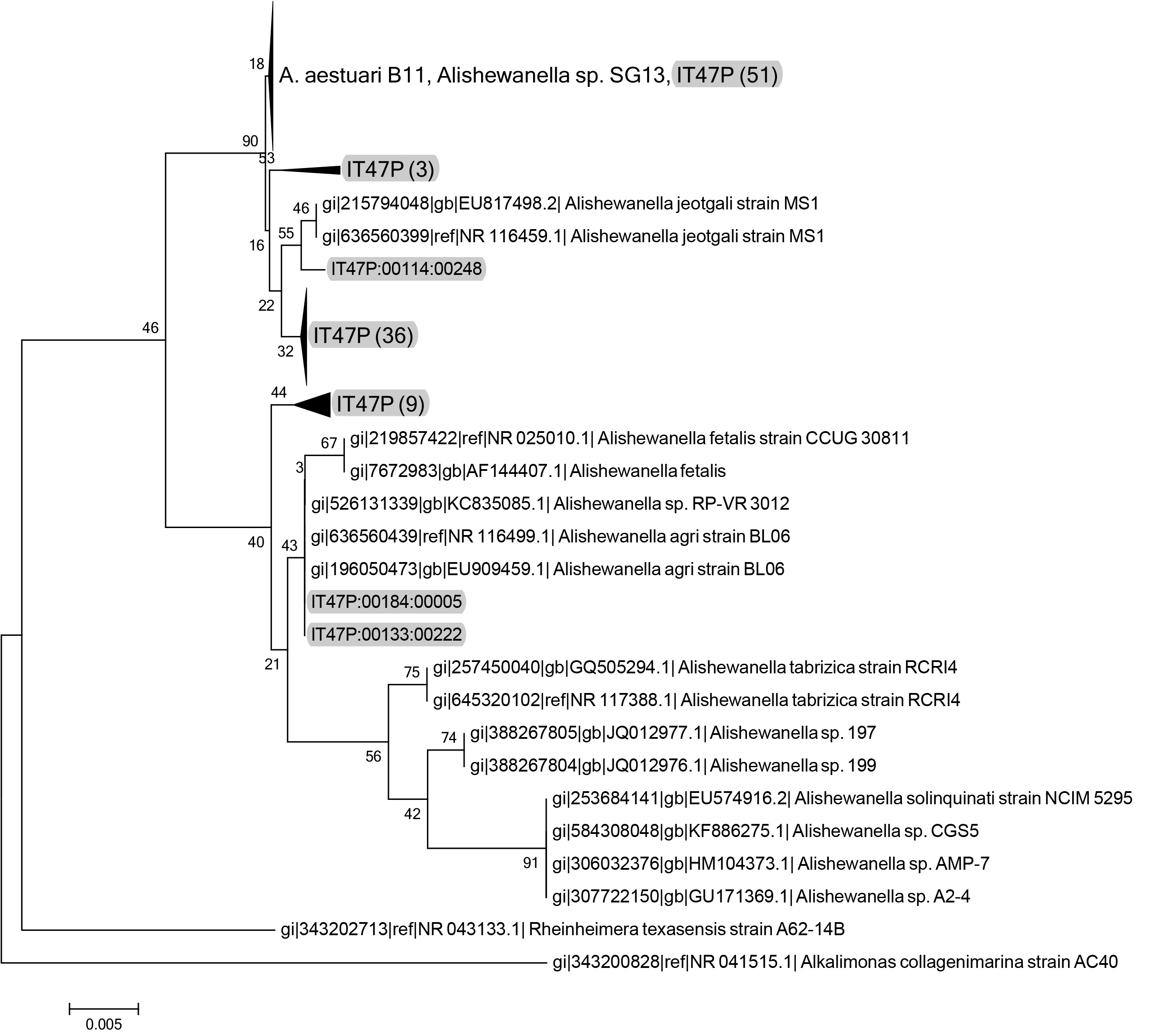

Supplement: Figure S3 — A genus level phylogenetic tree of Alishewanella reads. The tree was constructed using Neighbor-Joining algorithm. Rheinheimera and Alkalimonas were used as out-group. 102 Alishewanella reads were randomly selected from sample 47P and aligned with Alishewanella reference sequences obtained from NCBI. The number in the parentheses represents the number of 47P Alishewanella reads in the respective clade. (TIF) [file pone.0107534.s003.tif]

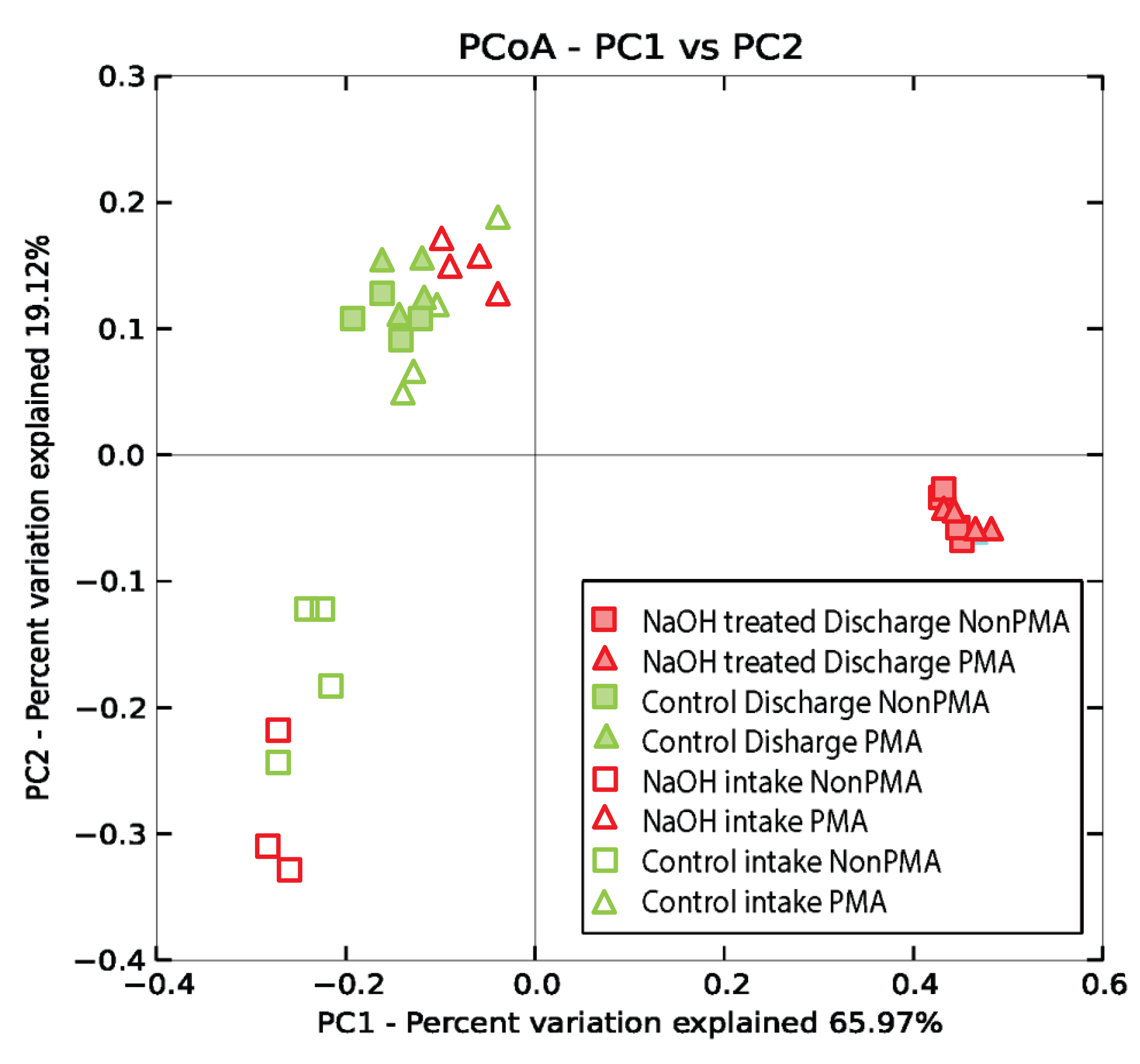

Supplement: Figure S4 — Principal coordinate analysis (PCoA) plots of the ballast water samples using weighted UniFrac distance. (TIF) [file pone.0107534.s004.tif]

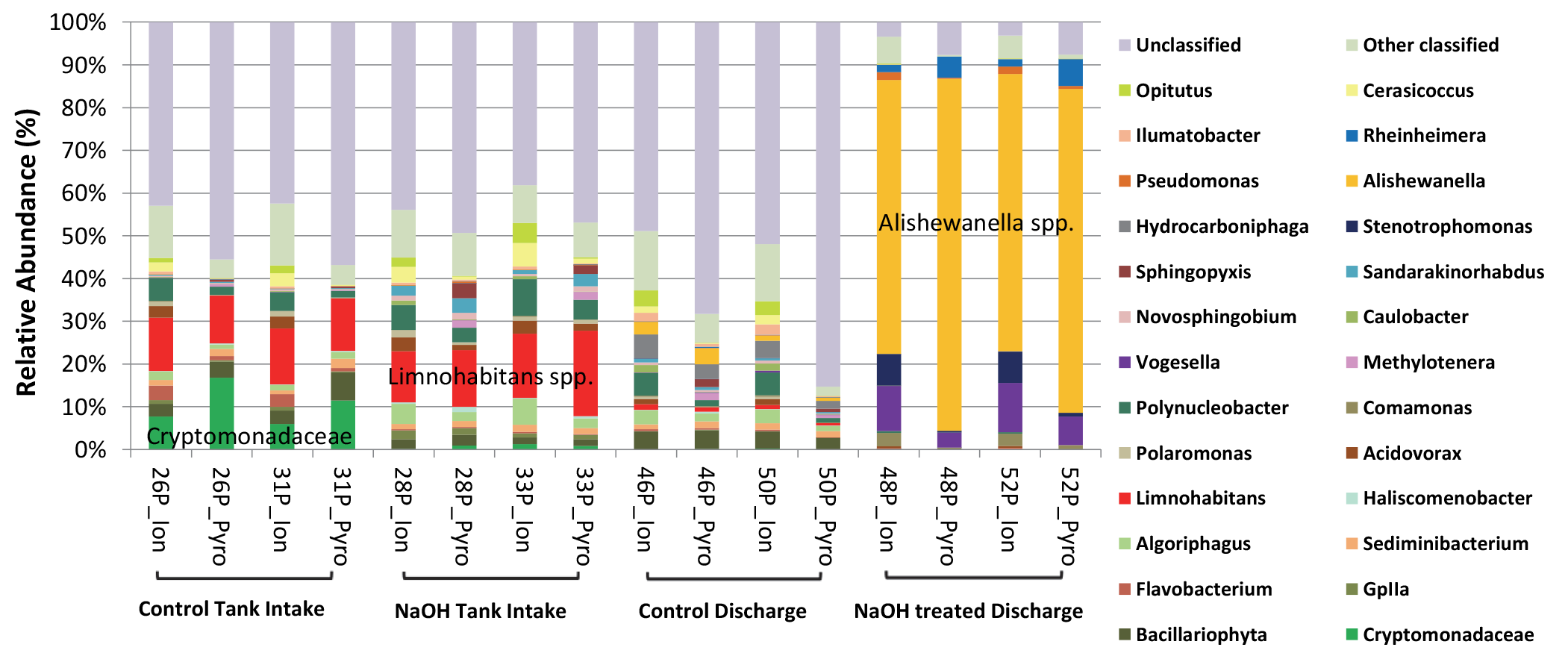

Supplement: Figure S5 — Comparison of microbial community assembly derived from Ion Torrent and pyrosequencing. RDP multiclassifier with 60% threshold was used to identify microbial taxa in each sample at genus level. Taxa that had the relative abundance of 1.5% or greater at least one of the samples were included in this figure. (TIF) [file pone.0107534.s005.tif]
