# Supplementary material for: Application of Ion Torrent Sequencing to the Assessment of the Effect of Alkali Ballast Water Treatment on Microbial Community Diversity
Source: PLoS One. 2014 Sep 15;9(9):e107534. doi: 10.1371/journal.pone.0107534 (PMC4164647; doi:10.1371/journal.pone.0107534)
Supplement: Table S1 — The number of reads and the average size obtained using Ion Torrent sequencing with the 400 chemistry for the 31 ballast water samples. (PDF) [file pone.0107534.s006.pdf]

Table S1: The number of reads and the average size obtained using Ion Torrent sequencing with the 400 chemistry for the 31 ballast water samples.

Sample ID	Ion PGM Run	Raw Reads	RDP Filtered Reads	Average Size
25P	First	19297	13136	378
26P	First	16774	11377	380
27P	First	17307	11675	379
28P	First	25342	17228	378
30P	First	18383	12228	379
31P	First	24800	16428	379
32P	First	23178	15542	378
33P	First	27276	18415	380
45P	First	27592	18427	378
46P	First	17907	11407	377
47P	First	22859	18065	386
48P	First	21938	16154	382
49P	First	24356	16466	378
50P	First	23363	15109	377
51P	First	27880	21906	386
52P	First	27872	19951	381
35N	Second	20045	15124	380
36N	Second	19930	14687	379
37N	Second	27853	20850	379
38N	Second	28432	21112	380
40N	Second	35638	26115	378
41N	Second	33958	24849	378
43N	Second	24184	18025	379
53N	Second	35933	25649	377
54N	Second	30465	22361	378
55N	Second	24442	19260	384
56N	Second	23941	19013	383
57N	Second	34711	25363	377
58N	Second	34501	24596	377
59N	Second	30604	24575	383
60N	Second	32384	25512	383
	Average	25907	18729	380
	Standard deviation	5664	4610	3
