# Supplementary material for: Application of Ion Torrent Sequencing to the Assessment of the Effect of Alkali Ballast Water Treatment on Microbial Community Diversity
Source: PLoS One. 2014 Sep 15;9(9):e107534. doi: 10.1371/journal.pone.0107534 (PMC4164647; doi:10.1371/journal.pone.0107534)
Supplement: Table S2 — Summary of diversity measurements for each sample rarefied at 10918 reads. (PDF) [file pone.0107534.s007.pdf]

Table S2. Summary of diversity measurements for each sample rarefied at 10918 reads

Sample_ID	Timing	Treatment	Number of OTUs	Chao1	Shannon
37N	Intake	NaOH	984.2	1531.1	6.968
38N	Intake	NaOH	778.8	1197.0	6.332
43N	Intake	NaOH	674.9	1093.8	6.498
27P	Intake	NaOH	1076.5	1743.8	6.885
28P	Intake	NaOH	962.6	1533.4	7.076
32P	Intake	NaOH	1057.7	1785.4	7.006
33P	Intake	NaOH	815.8	1312.8	6.565
35N	Intake	Control	1075.3	1715.9	6.724
36N	Intake	Control	982.7	1538.0	6.983
40N	Intake	Control	1080.4	1604.6	7.332
41N	Intake	Control	1141	1784.8	7.354
25P	Intake	Control	743.4	1204.6	7.054
26P	Intake	Control	1284.2	2228.8	7.403
30P	Intake	Control	1086.3	1754.2	7.254
31P	Intake	Control	1414.4	2285.0	7.705
55N	Discharge	NaOH	366.4	626.4	2.716
56N	Discharge	NaOH	419	688.8	3.512
59N	Discharge	NaOH	395.2	638.8	2.995
60N	Discharge	NaOH	387.7	611.5	3.405
47P	Discharge	NaOH	304	475.0	2.004
48P	Discharge	NaOH	535.8	884.8	3.800
51P	Discharge	NaOH	283.9	515.5	1.602
52P	Discharge	NaOH	530.1	921.4	3.735
53N	Discharge	Control	1417.9	2401.0	7.938
54N	Discharge	Control	1284.5	2083.5	7.713
57N	Discharge	Control	1343.7	2143.8	8.062
58N	Discharge	Control	1216.9	1926.5	7.715
45P	Discharge	Control	1303.5	2122.1	7.885
46P	Discharge	Control	1236.8	1945.7	7.947
49P	Discharge	Control	1251.8	1976.7	7.752
50P	Discharge	Control	1362.8	2210.5	7.977
