# Supplementary material for: Application of Ion Torrent Sequencing to the Assessment of the Effect of Alkali Ballast Water Treatment on Microbial Community Diversity
Source: PLoS One. 2014 Sep 15;9(9):e107534. doi: 10.1371/journal.pone.0107534 (PMC4164647; doi:10.1371/journal.pone.0107534)
Supplement: Table S3 — Pearson correlation analysis for Biom matrix data. (PDF) [file pone.0107534.s008.pdf]

Supporting Information (Table S3): Pearson correlation analysis for Biom matrix data

	46P	49P	37N	43N	32P	58N	54N	57N	53N	41N	36N	38N	40N	50P	33P	45P	28P	51P	31P	30P	26P	35N	56N	48P	52P	25P	27P	60N	47P	55N	59N
46P	1.000	0.792	0.262	0.158	0.548	0.862	0.854	0.780	0.840	0.388	0.295	0.140	0.370	0.928	0.542	0.847	0.572	0.204	0.486	0.577	0.520	0.216	0.218	0.209	0.210	0.512	0.485	0.218	0.205	0.210	0.213
49P	0.792	1.000	0.245	0.144	0.444	0.743	0.700	0.743	0.800	0.299	0.218	0.122	0.305	0.820	0.492	0.934	0.493	0.008	0.347	0.472	0.373	0.181	0.014	0.013	0.013	0.474	0.354	0.014	0.009	0.010	0.012
37N	0.262	0.245	1.000	0.957	0.290	0.346	0.326	0.349	0.354	0.924	0.946	0.963	0.964	0.261	0.340	0.275	0.314	0.002	0.483	0.420	0.554	0.971	0.009	0.003	0.004	0.311	0.277	0.010	0.002	0.005	0.007
43N	0.158	0.144	0.957	1.000	0.158	0.231	0.210	0.248	0.233	0.870	0.916	0.977	0.934	0.163	0.192	0.169	0.176	0.003	0.368	0.287	0.444	0.980	0.006	0.003	0.004	0.192	0.149	0.007	0.003	0.004	0.005
32P	0.548	0.444	0.290	0.158	1.000	0.539	0.561	0.545	0.624	0.510	0.424	0.165	0.403	0.487	0.950	0.517	0.968	0.003	0.936	0.964	0.913	0.223	0.039	0.012	0.015	0.818	0.980	0.043	0.003	0.017	0.026
58N	0.862	0.743	0.346	0.231	0.539	1.000	0.978	0.858	0.918	0.480	0.383	0.224	0.468	0.934	0.533	0.839	0.559	0.133	0.503	0.593	0.557	0.291	0.147	0.137	0.138	0.506	0.478	0.148	0.133	0.139	0.142
54N	0.854	0.700	0.326	0.210	0.561	0.978	1.000	0.863	0.907	0.474	0.378	0.205	0.453	0.926	0.544	0.811	0.568	0.128	0.526	0.613	0.581	0.277	0.143	0.132	0.133	0.525	0.509	0.145	0.129	0.135	0.138
57N	0.780	0.743	0.349	0.248	0.545	0.858	0.863	1.000	0.917	0.445	0.370	0.241	0.436	0.842	0.546	0.850	0.603	0.021	0.498	0.604	0.542	0.293	0.033	0.024	0.025	0.522	0.491	0.033	0.022	0.026	0.028
53N	0.840	0.800	0.354	0.233	0.624	0.918	0.907	0.917	1.000	0.469	0.381	0.222	0.465	0.877	0.630	0.895	0.668	0.013	0.561	0.684	0.597	0.293	0.028	0.017	0.018	0.577	0.567	0.029	0.013	0.019	0.022
41N	0.388	0.299	0.924	0.870	0.510	0.480	0.474	0.445	0.469	1.000	0.976	0.881	0.951	0.385	0.530	0.353	0.505	0.001	0.702	0.624	0.762	0.907	0.019	0.006	0.007	0.480	0.504	0.021	0.001	0.009	0.013
36N	0.295	0.218	0.946	0.916	0.424	0.383	0.378	0.370	0.381	0.976	1.000	0.926	0.953	0.282	0.443	0.263	0.423	0.000	0.636	0.542	0.699	0.938	0.015	0.004	0.005	0.402	0.429	0.017	0.000	0.006	0.010
38N	0.140	0.122	0.963	0.977	0.165	0.224	0.205	0.241	0.222	0.881	0.926	1.000	0.931	0.146	0.196	0.148	0.177	0.001	0.381	0.295	0.457	0.976	0.005	0.001	0.002	0.197	0.160	0.006	0.001	0.003	0.004
40N	0.370	0.305	0.964	0.934	0.403	0.468	0.453	0.436	0.465	0.951	0.953	0.931	1.000	0.381	0.419	0.359	0.410	0.002	0.580	0.530	0.654	0.961	0.014	0.005	0.006	0.407	0.387	0.015	0.002	0.007	0.010
50P	0.928	0.820	0.261	0.163	0.487	0.934	0.926	0.842	0.877	0.385	0.282	0.146	0.381	1.000	0.469	0.899	0.503	0.088	0.422	0.522	0.476	0.220	0.099	0.093	0.093	0.476	0.407	0.099	0.088	0.092	0.095
33P	0.542	0.492	0.340	0.192	0.950	0.533	0.544	0.546	0.630	0.530	0.443	0.196	0.419	0.469	1.000	0.537	0.959	0.001	0.909	0.954	0.879	0.261	0.034	0.010	0.013	0.799	0.946	0.038	0.001	0.015	0.023
45P	0.847	0.934	0.275	0.169	0.517	0.839	0.811	0.850	0.895	0.353	0.263	0.148	0.359	0.899	0.537	1.000	0.572	0.017	0.418	0.549	0.454	0.214	0.025	0.019	0.020	0.519	0.423	0.025	0.017	0.019	0.022
28P	0.572	0.493	0.314	0.176	0.968	0.559	0.568	0.603	0.668	0.505	0.423	0.177	0.410	0.503	0.959	0.572	1.000	0.002	0.903	0.956	0.879	0.234	0.035	0.010	0.013	0.806	0.951	0.038	0.002	0.015	0.023
51P	0.204	0.008	0.002	0.003	0.003	0.133	0.128	0.021	0.013	0.001	0.000	0.001	0.002	0.088	0.001	0.017	0.002	1.000	0.002	0.002	0.001	0.000	0.988	0.984	0.986	0.004	0.002	0.989	1.000	0.998	0.996
31P	0.486	0.347	0.483	0.368	0.936	0.503	0.526	0.498	0.561	0.702	0.636	0.381	0.580	0.422	0.909	0.418	0.903	0.002	1.000	0.953	0.984	0.433	0.037	0.011	0.014	0.761	0.948	0.041	0.002	0.016	0.025
30P	0.577	0.472	0.420	0.287	0.964	0.593	0.613	0.604	0.684	0.624	0.542	0.295	0.530	0.522	0.954	0.549	0.956	0.002	0.953	1.000	0.939	0.361	0.036	0.011	0.013	0.807	0.965	0.040	0.002	0.016	0.024
26P	0.520	0.373	0.554	0.444	0.913	0.557	0.581	0.542	0.597	0.762	0.699	0.457	0.654	0.476	0.879	0.454	0.879	0.001	0.984	0.939	1.000	0.508	0.035	0.010	0.012	0.760	0.916	0.039	0.001	0.015	0.023
35N	0.216	0.181	0.971	0.980	0.223	0.291	0.277	0.293	0.293	0.907	0.938	0.976	0.961	0.220	0.261	0.214	0.234	0.000	0.433	0.361	0.508	1.000	0.006	0.001	0.002	0.249	0.216	0.007	0.000	0.003	0.004
56N	0.218	0.014	0.009	0.006	0.039	0.147	0.143	0.033	0.028	0.019	0.015	0.005	0.014	0.099	0.034	0.025	0.035	0.988	0.037	0.036	0.035	0.006	1.000	0.994	0.995	0.032	0.040	1.000	0.990	0.994	0.996
48P	0.209	0.013	0.003	0.003	0.012	0.137	0.132	0.024	0.017	0.006	0.004	0.001	0.005	0.093	0.010	0.019	0.010	0.984	0.011	0.011	0.010	0.001	0.994	1.000	1.000	0.013	0.013	0.995	0.986	0.989	0.990
52P	0.210	0.013	0.004	0.004	0.015	0.138	0.133	0.025	0.018	0.007	0.005	0.002	0.006	0.093	0.013	0.020	0.013	0.986	0.014	0.013	0.012	0.002	0.995	1.000	1.000	0.014	0.015	0.995	0.988	0.991	0.992
25P	0.512	0.474	0.311	0.192	0.818	0.506	0.525	0.522	0.577	0.480	0.402	0.197	0.407	0.476	0.799	0.519	0.806	0.004	0.761	0.807	0.760	0.249	0.032	0.013	0.014	1.000	0.784	0.034	0.005	0.017	0.024
27P	0.485	0.354	0.277	0.149	0.980	0.478	0.509	0.491	0.567	0.504	0.429	0.160	0.387	0.407	0.946	0.423	0.951	0.002	0.948	0.965	0.916	0.216	0.040	0.013	0.015	0.784	1.000	0.044	0.003	0.018	0.027
60N	0.218	0.014	0.010	0.007	0.043	0.148	0.145	0.033	0.029	0.021	0.017	0.006	0.015	0.099	0.038	0.025	0.038	0.989	0.041	0.040	0.039	0.007	1.000	0.995	0.995	0.034	0.044	1.000	0.990	0.995	0.996
47P	0.205	0.009	0.002	0.003	0.003	0.133	0.129	0.022	0.013	0.001	0.000	0.001	0.002	0.088	0.001	0.017	0.002	1.000	0.002	0.002	0.001	0.000	0.990	0.986	0.988	0.005	0.003	0.990	1.000	0.999	0.997
55N	0.210	0.010	0.005	0.004	0.017	0.139	0.135	0.026	0.019	0.009	0.006	0.003	0.007	0.092	0.015	0.019	0.015	0.998	0.016	0.016	0.015	0.003	0.994	0.989	0.991	0.017	0.018	0.995	0.999	1.000	0.999
59N	0.213	0.012	0.007	0.005	0.026	0.142	0.138	0.028	0.022	0.013	0.010	0.004	0.010	0.095	0.023	0.022	0.023	0.996	0.025	0.024	0.023	0.004	0.996	0.990	0.992	0.024	0.027	0.996	0.997	0.999	1.000
