# Supplementary material for: Application of Ion Torrent Sequencing to the Assessment of the Effect of Alkali Ballast Water Treatment on Microbial Community Diversity
Source: PLoS One. 2014 Sep 15;9(9):e107534. doi: 10.1371/journal.pone.0107534 (PMC4164647; doi:10.1371/journal.pone.0107534)
Supplement: Table S4 — Pearson correlation analysis for the RDP multiclassifier output. (PDF) [file pone.0107534.s009.pdf]

Supporting Information (Table S4): Pearson correlation analysis for the RDP multiclassifier output

	26P_Ion	26P_Pyro	28P_Ion	28P_Pyro	31P_Ion	31P_Pyro	33P_Ion	33P_Pyro	46P_Ion	46P_Pyro	48P_Ion	48P_Pyro	50P_Ion	50P_Pyro	52P_Ion	52P_Pyro
26P_Ion	1.000	0.851	0.827	0.810	0.986	0.899	0.841	0.831	0.406	0.311	-0.006	-0.009	0.437	0.345	-0.007	-0.009
26P_Pyro	0.851	1.000	0.493	0.586	0.797	0.957	0.520	0.583	0.189	0.237	-0.006	-0.006	0.204	0.261	-0.006	-0.006
28P_Ion	0.827	0.493	1.000	0.883	0.861	0.625	0.970	0.895	0.533	0.355	-0.007	-0.009	0.572	0.406	-0.007	-0.009
28P_Pyro	0.810	0.586	0.883	1.000	0.836	0.720	0.837	0.973	0.339	0.404	-0.008	-0.009	0.348	0.430	-0.008	-0.009
31P_Ion	0.986	0.797	0.861	0.836	1.000	0.871	0.872	0.865	0.406	0.301	-0.007	-0.009	0.435	0.330	-0.007	-0.009
31P_Pyro	0.899	0.957	0.625	0.720	0.871	1.000	0.632	0.715	0.277	0.362	-0.007	-0.006	0.292	0.410	-0.007	-0.007
33P_Ion	0.841	0.520	0.970	0.837	0.872	0.632	1.000	0.872	0.549	0.313	-0.004	-0.008	0.590	0.368	-0.005	-0.008
33P_Pyro	0.831	0.583	0.895	0.973	0.865	0.715	0.872	1.000	0.296	0.296	-0.006	-0.007	0.297	0.320	-0.006	-0.007
46P_Ion	0.406	0.189	0.533	0.339	0.406	0.277	0.549	0.296	1.000	0.753	0.251	0.249	0.966	0.751	0.249	0.248
46P_Pyro	0.311	0.237	0.355	0.404	0.301	0.362	0.313	0.296	0.753	1.000	0.460	0.466	0.684	0.932	0.460	0.465
48P_Ion	-0.006	-0.006	-0.007	-0.008	-0.007	-0.007	-0.004	-0.006	0.251	0.460	1.000	0.985	0.118	0.172	1.000	0.989
48P_Pyro	-0.009	-0.006	-0.009	-0.009	-0.009	-0.006	-0.008	-0.007	0.249	0.466	0.985	1.000	0.109	0.174	0.984	0.999
50P_Ion	0.437	0.204	0.572	0.348	0.435	0.292	0.590	0.297	0.966	0.684	0.118	0.109	1.000	0.735	0.117	0.110
50P_Pyro	0.345	0.261	0.406	0.430	0.330	0.410	0.368	0.320	0.751	0.932	0.172	0.174	0.735	1.000	0.172	0.173
52P_Ion	-0.007	-0.006	-0.007	-0.008	-0.007	-0.007	-0.005	-0.006	0.249	0.460	1.000	0.984	0.117	0.172	1.000	0.989
52P_Pyro	-0.009	-0.006	-0.009	-0.009	-0.009	-0.007	-0.008	-0.007	0.248	0.465	0.989	0.999	0.110	0.173	0.989	1.000
